# Pregnancy Outcomes in Women With Vascular Ehlers-Danlos Syndrome: A Systematic Review of Maternal and Fetal Complications

**DOI:** 10.7759/cureus.114336

**Published:** 2026-08-11

**Authors:** Shaikha Almheiri, Asma ElMubarak, Omsalama Fadlalsaed

**Affiliations:** 1 Department of Obstetrics and Gynecology, Tawam Hospital, Abu Dhabi, ARE; 2 Department of Obstetrics and Gynecology, Danat Al Emarat Hospital, Abu Dhabi, ARE

**Keywords:** eds, fetal complications, maternal mortality, uterine rupture, veds

## Abstract

The Ehlers-Danlos syndromes (EDS) comprise a clinically and genetically heterogeneous group of heritable connective tissue disorders marked by skin hyperextensibility, joint hypermobility, and tissue fragility. Vascular EDS (vEDS) is an autosomal dominant disorder caused by pathogenic variants in COL3A1, the gene encoding type III collagen. The diagnosis of vEDS is confirmed by identifying a pathogenic variant in COL3A1. This systematic review was conducted according to the PRISMA guidelines. Major outcomes included maternal, fetal, and neonatal complications in EDS pregnancies. A comprehensive search was conducted using electronic databases, including PubMed, Cochrane Library, and ScienceDirect, from inception to April 2026. vEDS showed the highest risk profile, with maternal mortality ranging between 0.10% and 50%; however, this wide range is attributed to the inclusion of old studies, in which recent advancements in genetic testing and fetal genotyping were not used. The most common reported obstetric complications were preeclampsia, postpartum hemorrhage, and severe perineal tears. Premature rupture of membranes and preterm premature rupture of membranes were strongly associated with fetal EDS. Preterm birth is the most common complication, ranging between 8.9% and 48.8% of pregnancies across the included studies. Fetal EDS and COL3A1 mutations were more commonly associated with pregnancy complications than the maternal disease itself. Pregnancies complicated by EDS are associated with maternal, fetal, and neonatal complications. vEDS is associated with an increased risk of maternal mortality and a high prevalence of preterm birth. This systematic review underscores the significance of fetal EDS status and COL3A1 variants as a strong predictor of EDS pregnancy complications.

## Introduction and background

The Ehlers-Danlos syndromes (EDS) are defined as a clinically and genetically heterogeneous group of heritable connective tissue disorders, which are characterized by skin hyperextensibility, joint hypermobility, and tissue fragility [1]. EDS is classified into 13 clinical subtypes, which include classical EDS, classical-like EDS, cardiac-valvular EDS, vascular EDS (vEDS), hypermobile EDS, arthrochalasia EDS, and dermatosparaxis EDS [1]. vEDS, which was previously known as EDS type IV, is a dominantly inherited genetic disorder resulting from a genetic mutation in COL3A1, which is the gene responsible for type III collagen coding [2].

The initial diagnosis is based on family history and clinical history that often includes dissection or aneurysms, rupture of the large intestine, arterial rupture, and pregnancy complications at young ages [2]. These clinical signs and symptoms may overlap with other syndromes, such as Marfan syndrome, Loeys-Dietz syndrome, and familial arterial aneurysm and dissection syndromes. Therefore, a definitive diagnosis of vEDS is confirmed through identifying pathogenic variants in COL3A1 [2]. Type III collagen is an essential protein in the walls of blood vessels and hollow organs. The increased incidence of bruising and the fragility of several organs, such as the vagina, bowel, arteries, uterus, and cervix, are attributed to the genetic mutation of COL3A1 [3]. The abnormalities in type III collagen, in addition to the genetic mutation of COL3A1, result in vascular wall weakness, hence causing spontaneous rupture of medium-caliber and large-caliber vessels and the rupture of the uterus [2,3].

vEDS leads to several complications; however, the most common cause of death in women of childbearing age is complications related to it. For instance, vEDS is associated with pregnancy death in 5% of women. Additionally, the first pregnancies account for 50% of these pregnancy deaths associated with vEDS. The majority of mortality often results from non-pregnancy-specific complications, such as arterial complications. On the other hand, pregnancy-specific complications are rare and include uterine rupture during labor, premature rupture of membranes (PROM) with preterm delivery, and severe perineal tears, in addition to antepartum and postpartum hemorrhage [4].

A systematic review is crucial to extensively assess the current evidence, identifying pregnancy outcomes in women with vEDS, and evaluating the strengths and limitations of the existing literature. This review aims to synthesize evidence on pregnancy outcomes in women with vEDS and associated maternal and fetal complications.

## Review

Methods

Reporting Guidelines

The results were reported according to the PRISMA guidelines.

Outcome Definition and Eligibility Criteria

The search strategy of this systematic review was developed based on the Population, Intervention, Comparison, and Outcomes (PICO) framework and the study designs. Major outcomes of interest included maternal and fetal outcomes. The maternal outcomes included maternal mortality, arterial rupture/dissection, uterine rupture, postpartum hemorrhage, perineal tears, and pregnancy complications. The fetal outcome included miscarriage, preterm birth, stillbirth, neonatal mortality, and low birth weight. The target population was pregnant women diagnosed with vEDS. Studies involving mixed EDS subtypes were also included if they reported pregnancy outcomes relevant to vEDS or provided clinically meaningful data that contributed to the understanding of maternal and fetal outcomes in pregnancies complicated by EDS. Eligible interventions comprised the presence of vEDS (genetically or clinically diagnosed). Comparator groups included the general obstetric population and other EDS subtypes. The included study designs were observational studies, including cohort, case-control, cross-sectional, and case series.

Single case reports, editorials, commentaries, expert opinions without primary data, conference abstracts without full text, and narrative reviews were excluded. Additionally, studies with insufficient or unclear data regarding outcomes, laboratory studies, posters, protocols, and articles not published in English were excluded as well.

Information Sources

A comprehensive search was conducted using electronic databases, including PubMed, Cochrane Library, and ScienceDirect, from inception to April 2026. The search strategy employed a combination of keywords and Medical Subject Headings (MeSH) related to the PICO framework. Keywords or search terms were combined using Boolean operators (AND/OR) to capture relevant studies.

Search Strategy

The search strategies used for each database are presented below:

PubMed: (("Ehlers-Danlos Syndrome, Vascular"[Mesh] OR "vascular Ehlers-Danlos syndrome" OR "vEDS" OR "Ehlers-Danlos syndrome type IV" OR "COL3A1") AND ("Pregnancy"[Mesh] OR pregnancy OR pregnant OR gestation OR obstetric*) AND ("Pregnancy Outcome"[Mesh] OR "Pregnancy Complications"[Mesh] OR "Maternal Mortality"[Mesh] OR "Fetal Outcome" OR "perinatal outcome*" OR miscarriage OR "preterm birth" OR "premature birth" OR stillbirth OR "neonatal outcome*" OR "postpartum hemorrhage" OR "uterine rupture" OR "vascular complications"))

Cochrane: (("vascular Ehlers-Danlos syndrome" OR vEDS OR "Ehlers-Danlos type IV" OR COL3A1) AND (pregnancy OR pregnant OR gestation OR obstetric*) AND ("pregnancy outcome" OR "maternal outcome" OR "fetal outcome" OR miscarriage OR "preterm birth" OR stillbirth OR "maternal mortality" OR "uterine rupture" OR "postpartum hemorrhage")):ti,ab,kw" (Word variations have been searched)

ScienceDirect: ("vascular Ehlers-Danlos syndrome" OR "Ehlers-Danlos type IV") AND (pregnancy OR pregnant) AND (outcomes OR complications OR "pregnancy outcome" OR miscarriage OR "preterm birth" OR stillbirth)

Study Selection and Data Extraction

All records identified through the database search were imported into reference management software (EndNote X8, Clarivate Analytics, Philadelphia, PA, USA), and duplicates were removed. Two reviewers independently screened titles and abstracts for eligibility. Full-text articles were then retrieved and assessed independently by the same reviewers against the predefined inclusion and exclusion criteria. Disagreements at any stage of the screening process were resolved through discussion and, when necessary, consultation with a third reviewer. The study selection process was documented using a PRISMA flow diagram.

Data were independently extracted by two reviewers using a standardized data extraction form. Extracted information included study characteristics (author, year, country, and study design), participant characteristics (type of vEDS or EDS of the study population), and key findings related to maternal complications, fetal or neonatal complications, and the predictors or risk factors for adverse outcomes. Any discrepancies in data extraction were resolved by consensus.

Quality Assessment

The methodological quality and risk of bias of the included studies were assessed using established assessment tools. The Newcastle-Ottawa Scale (NOS) was used for observational studies, including cohort and case-control designs [5]. The assessment focused on methodological aspects such as study design, sample size, data collection methods, and risk of bias.

Results

Search Results

**Figure 1:**
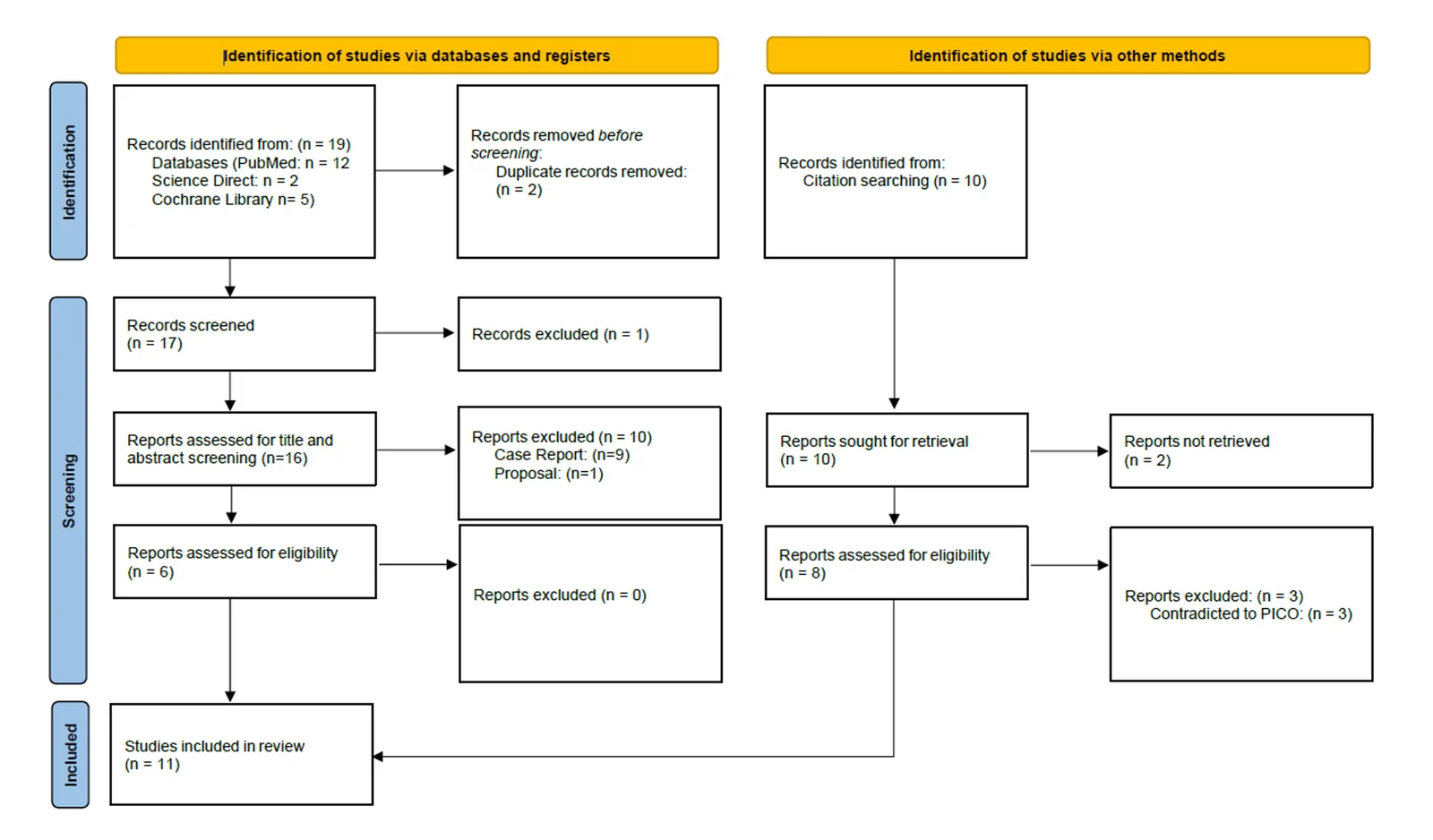
PRISMA flow chart.

The initial search found 19 articles. After removing duplicates and screening titles, abstracts, and full texts, the search was narrowed to 11 studies. Figure 1 shows the study selection process.

Characteristics of the Included Studies

Eleven studies met the inclusion criteria, published between 1983 and 2026 in different countries such as the USA, France, the Netherlands, and Canada [6-16]. All the studies included were observational studies. Most study designs were retrospective observational studies, with a few prospective study designs. The included studies collectively evaluated maternal and fetal outcomes in women with vEDS and the associated risk factors and predictors. Sample sizes ranged from six pregnancies in women with confirmed or suspected vEDS to large population-based cohorts involving 14,513,587 pregnant women. Table 1 summarizes the key details of the studies included in this systematic review.

**Table 1:** Baseline characteristics of the included studies. EDS, Ehlers-Danlos syndrome; ROPAC, Registry of Pregnancy and Cardiac Disease; vEDS, vascular Ehlers-Danlos syndrome

Author (year)	Country/setting	Study design	Population/sample size	vEDS/EDS type	Study objective
Dubruc et al. (2013) [6]	France	Retrospective observational study	27 pregnancies	vEDS (EDS type IV)	To describe obstetric care and pregnancy-related complications in women with vEDS
Hurst et al. (2014) [7]	USA	Prospective online questionnaire survey	775 reproductive-aged women with typed EDS diagnosis	Mixed EDS subtypes, including vascular type	To evaluate obstetric and gynecologic complications among women with EDS
Lind and Wallenburg (2002) [8]	Netherlands	Retrospective cohort study	168 EDS patients	Mixed EDS types, including type IV	To assess pregnancy outcomes and maternal/neonatal complications in EDS
Murray et al. (2014) [9]	USA	Retrospective pedigree review and semi-structured interview study	565 deliveries overall; detailed interviews from 39 women	vEDS	To characterize pregnancy-related mortality and morbidity in women with vEDS
Pepin et al. (2000) [10]	USA	Clinical and genetic cohort study	220 index patients and 199 affected relatives	vEDS (COL3A1-confirmed)	To describe clinical manifestations and complications of vEDS
Peters et al. (2026) [11]	International/ROPAC III registry	Prospective case series	6 pregnancies in women with confirmed or suspected vEDS	vEDS	To evaluate pregnancy outcomes and aortic changes in women with vEDS
Rudd et al. (1983) [12]	Canada	Family-based observational study	20 women with EDS IV	EDS type IV (vascular type)	To investigate pregnancy complications in women with vEDS
Spiegel et al. (2022) [13]	USA	Population-based retrospective cohort study	14,513,587 women admitted for delivery in US hospitals (1999-2014), including 1,042 deliveries in women with EDS	EDS overall	To evaluate maternal and neonatal outcomes associated with EDS pregnancies
Stephens et al. (2022) [14]	USA	Cohort study	41 children with pathogenic/likely pathogenic COL3A1 variants	vEDS	To evaluate perinatal and neonatal outcomes in children with vEDS
Underhill et al. (2022) [15]	USA	Web-based questionnaire study	EDS mother/EDS infant (n = 559), EDS mother/non-EDS infant (n = 304), and non-EDS mother/EDS infant (n = 91)	Mixed EDS types	To compare maternal versus fetal contribution to adverse pregnancy outcomes
van den Bersselaar et al. (2026) [16]	Netherlands / Multicenter	Retrospective multicenter cohort study	121 pregnancies in 43 women	vEDS with pathogenic/likely pathogenic COL3A1 variants	To evaluate pregnancy and delivery outcomes in women with vEDS

Outcome Measures of the Included Studies

The included studies evaluated maternal, fetal, and neonatal outcome measures in pregnancies complicated by EDS. The maternal outcomes included aortic and arterial rupture, maternal mortality, uterine rupture, postpartum hemorrhage, placental abnormalities, such as placenta previa and placental abruption, preeclampsia, thromboembolic events, cesarean section complications, severe perineal lacerations, PROM/preterm premature rupture of membranes (PPROM), and obstetric complications. Additionally, some studies evaluated gynecologic and reproductive complications, such as miscarriage, infertility, dysmenorrhea, dyspareunia, and ectopic pregnancies. Fetal and neonatal outcomes included spontaneous abortion, congenital anomalies, intrauterine growth restriction, preterm birth, intrauterine fetal death, low birth weight, stillbirth, neonatal deaths, and neonatal complications, such as intracranial hemorrhage and pneumothorax. Multiple studies investigated predictors of adverse outcomes and risk factors, including EDS subtype, genotype-specific variants, prior vascular history, fetal EDS status, COL3A1 mutations, cervical shortening, and twin pregnancy.

The findings of the included studies reported that maternal mortality estimates varied significantly according to study design and sample size, with a large population-based cohort reporting a low risk of 0.10%, whereas studies with small sample sizes reported a substantially higher mortality rate (33-50%). Moderate-sized cohort studies reported a medium range of mortality rates ranging between 6.5% and 14.8%, suggesting that the highest mortality rates are affected by the small sample size [9,10,12,14]. The most common reported obstetric complications were preeclampsia, postpartum hemorrhage, severe perineal tears, placenta previa, thromboembolism, placental abruption, cesarean complications, and cervical incompetence [8,9,11,13,16]. Reproductive morbidity in patients with EDS was also evaluated by Hurst et al. [7] and included infertility, dyspareunia, gynecological surgical interventions, ectopic pregnancies, dysmenorrhea, and recurrent spontaneous abortion. Some moderate-sized cohorts reported that PROM and PPROM were strongly associated with fetal EDS [8,15,16] (Table 2).

**Table 2:** Maternal and fetal/neonatal outcomes in pregnancies complicated by EDS across included studies. BW, birth weight; CHD, congenital heart disease; EDS, Ehlers-Danlos syndrome; IUGR, intrauterine growth restriction; IUFD, intrauterine fetal death; PPROM, preterm premature rupture of membranes; PROM, premature rupture of membranes; PTB, preterm birth; SUA, single umbilical artery; vEDS, vascular Ehlers-Danlos syndrome; VSD, ventricular septal defect; VTE, venous thromboembolism

Author (year)	Maternal complications	Fetal/neonatal complications	Predictors/risk factors for adverse outcomes	Key conclusions
Hurst et al. (2014) [7]	Infertility (44.1%); spontaneous abortion (57.2%); ectopic pregnancy (5.1%); heavy menstrual bleeding (51.0%); irregular cycles (46.7%); intermenstrual bleeding (18.6%); dysmenorrhea (92.5%); dyspareunia (77.0%); gynecologic surgeries for ovarian cysts (14.1%), endometriosis (22.9%), adhesions (7.1%), uterine polyps (2.56%)	Preterm birth (25.2%); spontaneous abortion (<20 weeks: 57.2%); ectopic pregnancy (5.1%); term birth reduced in vEDS (57.7%)	EDS subtype strongly predictive: vEDS associated with higher PTB, bleeding, uterine pathology, and counseling against pregnancy; hypermobility EDS linked to infertility; classical EDS had better term outcomes	Significant reproductive morbidity in EDS with strong subtype-specific variation; vEDS carries highest obstetric risk burden
Lind and Wallenburg (2002) [8]	Postpartum hemorrhage (19%); severe perineal tears (8%); PROM (54.35%); preeclampsia (15.22%); pelvic instability (26%)	Preterm birth (22%); fetal death (4.35%); abnormal fetal presentation (12%); congenital anomalies (26.09%) (clubfoot, VSD, and spina bifida)	Fetal EDS status strongly associated with PROM and PTB; type IV EDS linked to severe vascular complications; twin pregnancy increased adverse outcomes	EDS pregnancies associated with high maternal and neonatal morbidity; fetal genotype significantly influences PTB and membrane rupture risk
Murray et al. (2014) [9]	Maternal mortality (6.5%); arterial rupture/dissection (9.2%); uterine rupture (2.6%); placental abruption (2.6%); placenta previa (3.9%); postpartum hemorrhage (10.5%); surgical complications (2.6%), third-/fourth-degree perineal lacerations (20%)	Preterm birth (19%); spontaneous abortion	Protein-altering COL3A1 mutations increased risk; null variants showed milder phenotype; vaginal delivery associated with lacerations; cesarean associated with surgical complications	vEDS carries high maternal mortality risk driven by arterial rupture; genotype influences severity of outcomes
Pepin et al. (2000) [10]	Maternal death in pregnancy (14.8%); arterial rupture/dissection (64.92%;272 cases overall); uterine rupture; bowel rupture; postpartum vascular rupture	Live births (91.2%); stillbirth (0.16%); spontaneous abortion (0.54%); preterm birth (12.4%); congenital anomalies (clubfoot and hip dislocation)	COL3A1 mutation confirmed disease cause; no clear genotype-severity correlation; risk increases with age (up to 80% by 40 years)	vEDS associated with extreme maternal mortality and vascular complications; pregnancy substantially increases fatal risk
Peters et al. (2026) [11]	Postpartum hemorrhage (16.7%); thromboembolism (16.7%); arterial dissection (16.7%); preeclampsia (16.7%); no maternal death (0%)	Preterm birth (33.33%); stillbirth (16.7%); neonatal pneumothorax (16.7%); birth weights 2320-3380 g	COL3A1 protein-altering variants; prior vascular history; cervical shortening; PPROM; stable aortic diameter not predictive of complications	No maternal mortality or aortic progression observed, but fetal loss and obstetric complications remain significant
Rudd et al. (1983) [12]	Maternal mortality (50%); uterine rupture; arterial rupture; bowel rupture; bladder rupture; severe postpartum hemorrhage; surgical complications	Neonatal death (16.67%; 1 case); congenital anomalies (clubfoot 16.67%; 1 case)	Type IV vEDS (COL3A1) major risk factor; pregnancy/labor identified as highest-risk period; no reliable predictors identified	Extremely high maternal mortality in vEDS; catastrophic vascular and uterine complications during pregnancy
Spiegel et al. (2022) [13]	Antepartum hemorrhage (2.59% vs 1.57%); placenta previa (1.44% vs 0.65%); cervical incompetence (1.92% vs 0.71%); CS (37.8% vs 27.5%); VTE (0.86% vs 0.32%); wound infection (0.38% vs 0.10%); maternal death (0.10% vs 0.01%)	Preterm birth (8.93% vs 6.53%); IUGR (3.36% vs 1.88%); IUFD (0.40% vs 0.58%)	Adjusted for age and race; smoking (11.0% vs 5.2%) and mitral valve prolapse higher in EDS; ICD-coded EDS exposure	EDS associated with increased maternal morbidity and neonatal complications; confirms high-risk obstetric classification
Stephens et al. (2022) [14]	Maternal mortality (33.3%); uterine rupture (1/6); cardiovascular collapse (1/6); placental abruption (1/6); aortic dissection (1/6); iliac artery rupture (1/6); CS common (66.7%)	Preterm birth (48.8%); low birth weight (26.8%); birth defects (41.5%), including CHD (14.6%), clubfoot (17.1%), hip dysplasia (7.3%)	Fetal COL3A1 genotype (glycine/splice variants) associated with prematurity and low BW; fetal genotype more predictive than maternal status	Fetal COL3A1 mutation is key determinant of prematurity and growth restriction; maternal complications remain severe
Underhill et al. (2022) [15]	Birth complications (80% in EDS mother/EDS infant group); CS (27-36%); miscarriage/stillbirth higher in EDS mothers; treatment for PTB (29.5%)	Preterm birth (30.9%-36.2% depending on group); PPROM higher in non-EDS mother/EDS infant group (38.9%); IUGR (5.5%)	Fetal EDS status stronger predictor than maternal status; PPROM significantly associated with fetal EDS (p = 0.025)	Fetal EDS drives adverse outcomes more than maternal EDS; PPROM and PTB significantly increased
van den Bersselaar et al. (2026) [16]	Severe perineal tears (18.6%); postpartum hemorrhage (14.0%); CS complications (hemorrhage, hysterectomy in isolated cases); no maternal death or uterine rupture	Congenital anomalies (3 cases): cleft lip/palate, SUA, clubfoot; intracranial hemorrhage (3 neonates); neurological disability in severe cases	Glycine substitution variants associated with higher miscarriage (19/23; p = 0.018); PPROM linked to PTB; fetal genotype key determinant of growth restriction	No maternal mortality or uterine rupture observed; however, obstetric morbidity remains high and fetal genotype strongly influences outcomes

Both fetal and neonatal outcomes were comparably unfavorable, with preterm birth being the most common complication, ranging between 8.9% and 48.8% of pregnancies across the included studies [7-9,11,13-15]. Moreover, neonatal complications included low birth weight, stillbirth, neonatal deaths, intracranial hemorrhage, intrauterine growth restriction, pneumothorax, and congenital anomalies, such as congenital heart defects, cleft lip or palate, spina bifida, hip dysplasia, and clubfoot [8,10,12,14,16] (Table 2).

Fetal EDS and COL3A1 mutations were identified as stronger predictors of fetal and obstetric complications, such as PROM, PPROM, and preterm birth, than the maternal EDS status alone [14,15]. Moreover, glycine substitution and protein-altering COL3A1 variants were associated with higher rates of growth restrictions, prematurity, miscarriage, and severe vascular complications [9,11,16]. On the other hand, maternal complications, such as uterine rupture, arterial rupture, and postpartum hemorrhage, were associated with maternal EDS status. Overall, the findings consistently support considering pregnancies complicated by EDS, especially vEDS, as high-risk pregnancies that require multidisciplinary management and individualized obstetric management (Table 2).

Quality Assessment Results

The methodological quality of the included observational studies was assessed using NOS, while the case series study was evaluated using the Joanna Briggs Institute (JBI) critical appraisal checklist for case series and analytical cross-sectional studies [17]. The included studies demonstrated moderate to good methodological quality, with four studies achieving the maximum score of 9 and classified as high-quality studies (Table 3). The case series demonstrated satisfactory methodological quality, fulfilling most appraisal criteria, including clear inclusion criteria, valid diagnostic methods, and comprehensive reporting of demographic, clinical, and outcome data (Table 4). Both cross-sectional studies were considered to have acceptable methodological quality despite the presence of some limitations regarding condition measurement and confounding control (Table 5).

**Table 3:** Risk of bias in observational studies according to NOS tool. NOS, Newcastle-Ottawa Scale

Study	Selection (4)	Comparability (2)	Outcome (3)	Total score (9)	Quality assessment
Lind and Wallenburg (2002) [8]	3	1	3	7	Good
Murray et al. (2014) [9]	4	2	3	9	Good
Pepin et al. (2000) [10]	4	1	3	8	Good
Rudd et al. (1983) [12]	3	1	2	6	Moderate
Spiegel et al. (2022) [13]	4	2	3	9	Good
Stephens et al. (2022) [14]	3	1	3	7	Good
van den Bersselaar et al. (2026) [16]	4	2	3	9	Good

**Table 4:** JBI critical appraisal checklist of case series study. JBI, Joanna Briggs Institute

JBI criteria	Peters et al. (2026) [11]
Were there clear criteria for inclusion in the case series?	Yes
Was the condition measured in a standard, reliable way for all participants included in the case series?	Yes
Were valid methods used for identification of the condition for all participants included in the case series?	Yes
Did the case series have consecutive inclusion of participants?	Unclear
Did the case series have complete inclusion of participants?	Yes
Was there clear reporting of the demographics of the participants in the study?	Yes
Was there clear reporting of the clinical information of the participants?	Yes
Were the outcomes or follow-up results of cases clearly reported?	Yes
Was there clear reporting of the presenting site(s)/clinic(s) demographic information?	Yes
Was statistical analysis appropriate?	Yes

**Table 5:** JBI critical appraisal checklist for analytical cross-sectional studies. JBI, Joanna Briggs Institute

Criteria	Hurst et al. (2014) [7]	Underhill et al. (2022) [15]
Were the criteria for inclusion clearly defined?	Yes	Yes
Were the study subjects and setting described in detail?	Yes	Yes
Was the exposure measured in a valid and reliable way?	Yes	Yes
Were objective, standard criteria used for measurement of the condition?	No	No
Were confounding factors identified?	Unclear	Yes
Were strategies to deal with confounding stated?	No	Yes
Were outcomes measured in a valid and reliable way?	Yes	Yes
Was appropriate statistical analysis used?	Yes	Yes

Data Synthesis

Due to substantial clinical and methodological heterogeneity among the included studies, including differences in study design, study populations, outcome definitions, and reporting methods, a quantitative meta-analysis was not considered appropriate. Therefore, the findings were synthesized using a qualitative (narrative) approach, with results summarized descriptively according to maternal, fetal, and neonatal outcomes.

Discussion

The synthesized evidence of the included studies of this systematic review indicates that pregnancies complicated by EDS were associated with increased maternal, fetal, and neonatal complications, with the severity of the complications varying according to the subtype of EDS and the genetic profile. vEDS was associated with the highest risk profile of maternal mortality, primarily due to arterial rupture, uterine rupture, aortic dissection, severe hemorrhage, and bowel rupture. Although vEDS was associated with high maternal mortality rates, the most recent studies reported lower rates of maternal mortality, which can be attributed to advances in multidisciplinary obstetric care and genetic diagnosis. Additionally, severe vascular complications were associated with EDS pregnancies, resulting in preeclampsia, severe perineal tears, PROM and PPROM, and placental abnormalities, which supports classifying EDS pregnancies as high-risk pregnancies. The presence of considerable heterogeneity in the sample size and study design of the included studies urges cautious interpretation of maternal outcomes, especially maternal mortality and vascular rupture.

Moreover, the synthesized evidence of the included studies identified the associated fetal and neonatal complications with EDS pregnancies, with preterm birth being the most reported adverse outcome, followed by low birth weight, stillbirth, intrauterine growth restrictions, congenital anomalies, and neonatal deaths. Additionally, fetal status and COL3A1 mutations were considered stronger predictors of pregnancy complications than the maternal disease itself. Glycine substitution and protein-altering COL3A1 variants were associated with higher rates of severe clinical phenotypes, such as growth restrictions, prematurity, miscarriage, and severe vascular complications, highlighting the significance of genetic counseling and molecular characterization in determining the expected pregnancy complications associated with EDS.

Maternal Outcomes

Pregnancy is well-tolerated in EDS patients with subtypes, such as classical EDS and hypermobile EDS. However, they can be associated with several maternal complications, such as perineal tearing, postpartum hemorrhage, pelvic prolapse, and incontinence following delivery [18-20]. Moreover, the abnormalities in collagen types I and V, which are associated with classic EDS, can significantly affect the cervix, causing cervical insufficiency, which can result in preterm birth [21]. vEDS is associated with type III collagen abnormalities, which result in vascular rupture, organ rupture, and fistula formation [2]. Therefore, during pregnancy, uterine contraction can result in uterine rupture and facilitate an increase in blood volume, heart rate, and blood pressure, increasing the risk of vessel rupture and dissection [22]. These factors render vEDS the most life-threatening subtype of EDS, with a 5% maternal death rate in deliveries [23].

There is a high prevalence of obstetric complications associated with vEDS pregnancy. We found that vEDS was associated with a high maternal mortality rate and high rates of obstetric complications, which is in line with the findings of other studies. For instance, Kang et al. [23] reported that third and fourth degrees of perineal tears were 20-fold more prevalent in vaginal deliveries in patients with vEDS, which can be attributed to the fragility of the skin and connective tissues. They reported that postpartum hemorrhage was more common in vEDS due to a greater risk of vascular rupture and higher rates of uterine rupture [23]. Haem et al. [24] reported in their systematic review that the maternal mortality rate per pregnancy was 5.7%, the miscarriage rate was 13.8%, and 8.8% of the live births were premature.

Fetal Outcomes

EDS causes fetal and neonatal complications, such as intrauterine growth restriction, low birth weight, stillbirth, neonatal deaths, intracranial hemorrhage, and congenital anomalies. We found that preterm birth was the most common fetal complication, ranging between 8.9% and 48.8% of pregnancies, in addition to fetal EDS acting as a strong predictor of pregnancy complications in EDS pregnancies. These findings agree with the findings of Kang et al. [23], who reported that the prevalence of preterm birth was 19% and is often associated with cervical insufficiency and PPROM. Moreover, it was found that prematurity and PROM were more common in fetuses with EDS in healthy mothers compared to fetuses without EDS in EDS-affected mothers, which highlights the significance of the fetal EDS status as a strong predictor of pregnancy complications [25]. Additionally, placental defects and abnormal placental vascular resistance in EDS patients resulted in intrauterine growth restriction [23].

This systematic review adds to the existing literature by synthesizing maternal, fetal, neonatal, and reproductive outcomes across women with different subtypes of EDS through incorporating recent studies that highlight current obstetric practice. This review underscores the correlation between EDS fetal status and COL3A1 mutations with the occurrence of maternal, fetal, and neonatal complications, further emphasizing that maternal and fetal EDS have distinct mechanisms and can result in different pregnancy complications. Additionally, it highlights the critical role of preconception genetic counseling and fetal genotyping in guiding clinical risk stratification.

Strengths and Limitations

This systematic review comprises several strengths, which include comprehensively evaluating maternal, fetal, and neonatal complications associated with EDS pregnancies across different study designs and evaluating subtype-specific risks and genotype-phenotype associations in predicting adverse outcomes. Additionally, evaluating methodological quality using validated tools, such as NOS and JBI critical appraisal checklists, enhances the reliability of the synthesized evidence. However, several limitations existed. Most of the included studies comprised a small sample size, which may limit generalizability. A considerable heterogeneity existed among the included studies regarding the diagnostic criteria, outcome definitions, and EDS subtype. The inclusion of older studies may have affected the rates of maternal mortality due to the recent advances in genetic testing and obstetric management. Additionally, the systematic review protocol was not registered, which limits transparency.

Future Directions

Future research is essential to produce a standardized diagnostic criterion to improve comparability. Additionally, investigating the correlation between COL3A1 variants and fetal EDS with pregnancy complications could enable the prediction of pregnancy outcome in EDS patients. Long-term follow-up studies are also essential to evaluate the extent of pregnancy complications on maternal health and the development of the child.

## Conclusions

Pregnancies complicated by EDS are associated with maternal, fetal, and neonatal complications. vEDS is associated with high maternal mortality rates and a high prevalence of preterm birth. Additionally, this systematic review underscores the significance of fetal EDS status and COL3A1 variants as a strong predictor of EDS pregnancy complications. Further studies are essential to fill the gap in the existing literature regarding the association between fetal EDS and COL3A1 mutations and pregnancy complications in EDS patients.
